# Extract of Wheatgrass and Aronia Mixture Ameliorates Atopic Dermatitis-Related Symptoms by Suppressing Inflammatory Response and Oxidative Stress In Vitro and In Vivo

**DOI:** 10.3390/antiox12010027

**Published:** 2022-12-23

**Authors:** Ji-Hyun Lee, Ji-Ye Lim, Yong-Deok Jeon, Dae-Ho Yun, Young-Mi Lee, Dae-Ki Kim

**Affiliations:** 1 Department of Immunology, Jeonbuk National University Medical School, Jeonju-si 54907, Republic of Korea; 2 Department of Korean Pharmacy, Woosuk University, Wanju-gun 55338, Republic of Korea; 3 Department of Health Administration, Kwangju Women’s University, Kwangju 62396, Republic of Korea; 4 Department of Oriental Pharmacy, College of Pharmacy, Wonkwang-Oriental Medicines Research Institute, Wonkwang University, Iksan 54538, Republic of Korea

**Keywords:** *Triticum aestivum* L., *Aronia melanocarpa*, atopic dermatitis, inflammation, oxidative stress

## Abstract

Atopic dermatitis is regulated by the production of pro-inflammatory cytokines and chemokines via the nuclear factor kappa B or mitogen-activated protein kinase signaling pathways, as well as, the release of oxidative stress-related factors via the NF-E2 p45-related factor 2 signaling pathway. Both wheatgrass (*Triticum aestivum* L., TA) and aronia (*Aronia melanocarpa*, AR) are known for their anti-inflammatory and antioxidant properties, however, the anti-inflammatory and antioxidant effects of TA and AR (TAAR) mixture extract have not been elucidated in an atopic dermatitis model. In this study, we assessed the inhibitory effects and underlying molecular mechanism of TAAR extract against lipopolysaccharide-induced inflammation and tumor necrosis factor-α/interferon-γ-induced inflammation and oxidative stress in vitro. We also investigated the alleviating effect of TAAR extract on DNCB-induced atopic dermatitis-like skin lesions in mice in vivo. We found that TAAR extract treatment inhibited inflammatory mediators in both RAW 264.7 cells and HaCaT cells, and increased the expression of oxidative stress defense enzymes in HaCaT cells. Furthermore, treatment of the DNCB-induced mouse model with TAAR extract ameliorated the overall symptoms of atopic dermatitis. Therefore, TAAR extract as a novel natural therapeutic agent may be used for the treatment of atopic dermatitis.

## 1. Introduction

Atopic dermatitis (AD), characterized by dry and sensitive skin, pruritus, and eczema, is a representative inflammatory skin disease that usually involves chronic relapse [1,2]. The prevalence of AD affects approximately 20% of children and adolescents and approximately 5% of adults worldwide, and its prevalence is increasing, especially in developing countries [3]. AD is considered a public health problem because it can significantly decrease a patient’s quality of life and inflict a financial burden on patients and the national healthcare system.

Although the pathogenesis of AD has not yet been fully elucidated, it is known to be caused by skin barrier abnormalities and immune dysfunction due to the complex action of genetic, environmental (e.g., mite dust, food allergen exposure, smoking), and immunological factors [4]. Currently, topical visible skin lesions anti-inflammatory drugs such as corticosteroids (e.g., glucocorticoids), calcineurin inhibitors (e.g., tacrolimus), and anti-histamine are commonly used to treat AD [5,6]. In addition, moisturizers that relieve xerosis and itching by increasing skin hydration and improving skin barrier function are widely used as adjuvant treatments [7]. Phototherapy and systemic immunosuppressants are also used for severe AD. Treatment drugs, such as corticosteroids or calcineurin inhibitors, can temporarily relieve the symptoms of AD by reducing inflammation, but long-term use may cause side effects such as local cutaneous atrophy, stinging, itching, telangiectasia, and burning sensation [8]. AD is easy to diagnose; however, despite the use of many therapeutic drugs, patients often relapse, and complete recovery is still not possible.

Recently, biological agents with good therapeutic effects have been developed, but their accessibility is limited owing to high prices that increase the financial burden on the patient’s family [9]. Therefore, there is a need to develop safe and effective therapeutic agents against AD with reasonable prices and few side effects. Hence, there has been a growing interest in the development of alternative therapeutic agents for the treatment of AD, especially natural bioactive compounds extracted from natural resources.

Wheat (*Triticum aestivum* L., TA) belonging to the Poaceae family is an important crop worldwide. The young grass of wheat, generally called wheatgrass (i.e., wheat sprout), is richer in nutrients than mature wheat grains because vitamins, polysaccharides, enzymes, proteins, and minerals are synthesized from wheat seeds during germination and sprouting [10]. In addition, wheatgrass is known for its high content of flavonoids and phenolic compounds and excellent antioxidant effects. Wheatgrass is currently used as a healthy food alternative in the form of juices, powders, and extracts. The anti-inflammatory, antioxidant [11], and anticancer effects [12] of wheatgrass has been shown before. Moreover, wheatgrass is also effective in the treatment of heart disease and colitis [13], as well as, alcoholic liver damage [14], LPS-induced liver injury [15], atopic dermatitis [16], allergies [17], and colorectal cancer [18].

*Aronia melanocarpa* (AR), a black chokeberry belonging to the Rosaceae family, was brought from North America to Eastern Europe, where it became more popular. Aronia is mainly consumed in the form of juice, fruit, wine, and jam and has also been used as a traditional medicine to cure several diseases [19]. Research has revealed that aronia contains a variety of nutrients and high levels of bioactive compounds, such as anthocyanins, polyphenols, and flavonoids [20]. Aronia has been used as a medicinal herb to treat hypertension and atherosclerosis, and in the pharmaceutical industry, aronia extract is used to produce health supplements [21]. The anti-inflammatory and antioxidant antidiabetic, antiviral, and immunomodulatory properties of aronia have been shown before [22,23,24].

To date, many studies have investigated the useful effects of wheatgrass and aronia, however, the therapeutic effect of TA and AR mixture extract (TAAR) in AD has not been investigated before. Therefore, in this study, we investigated the anti-inflammatory and antioxidant effects of wheatgrass and aronia mixture extract in vitro using RAW 264.7 macrophages and HaCaT human keratinocytes, as well as in vivo in a 2,4-dinitrochlorobenzene (DNCB)-induced mouse model of AD.

## 2. Materials and Methods

### 2.1. Chemicals and Reagents

Dulbecco’s modified Eagle’s medium (DMEM) and fetal bovine serum (FBS) were obtained from Gibco BRL (Invitrogen Co., Waltham, MA, USA). 3-(4,5-Dimethyl-2-thiazolyl)-2,5-diphenyl-2H-tetrazolium bromide (MTT), lipopolysaccharide (LPS), DNCB, and dexamethasone were purchased from Sigma-Aldrich. (St. Louis, MO, USA). Recombinant human tumor necrosis factor (TNF)-α and interferon (IFN)-γ were purchased from BioLegend (San Diego, CA, USA). Specific primary antibodies against inducible nitric oxide synthase (iNOS), cyclooxygenase-2 (COX-2), pp38, p38, pERK, ERK, pJNK, JNK, F4/80, Nrf2, heme oxygenase 1 (HO-1), NAD(P)H quinone dehydrogenase 1 (NQO1), pIκB-α, IκB-α, NFκB p65, lamin B, and β-actin, and secondary antibodies used for Western blotting and immunohistochemistry (IHC) were purchased from Santa Cruz (CA, USA), Abcam (Cambridge, UK), or Cell Signaling Technology (Danvers, MA, USA).

### 2.2. Preparation of TAAR Extract

TA was supplied by the National Institute of Crop Science and was cultivated at the Saemangeum Biotechnology Center (Gimje, Republic of Korea), and AR was imported from Poland. TA and AR were mixed in a ratio of 1:3, extracted twice using 30% ethanol eight times the total amount of TA and AR, at 60 °C for 4 h. After primary filtration, the extracts were concentrated at 600–700 mm/Hg and 60–70 °C, followed by sterilization at 95 °C for 60 min. After the secondary filtration, freeze-drying was performed. TAAR extract was obtained from Korea health food Corporation. The TAAR extract was stored at −20 °C, dissolved in purified water, and used for subsequent experiments. We mixed TA and AR in expectation of synergistic effects. As a result of preliminary experiments on the mixture of various ratios of TA and AR, it was confirmed that the ratio of 1:3 had excellent antioxidant and anti-inflammatory effects, and accordingly, the 1:3 mixture ratio was selected (data are not shown).

### 2.3. High Performance Liquid Chromatography (HPLC)

HPLC of the TAAR extract was performed on a Hitachi Chromaster (Hitachi, Japan) 5430 photodiode array (PDA) detector (Waters Corporation, Milford, MA, USA). The analytical column used was TSKgel ODS-100V (4.6 × 250 mm) (Tosoh, Japan). The column temperature was 25 °C, and the samples and injection volume was 10 mL. The mobile phase flow rate was 1.0 mL/min. The mobile phases were composed of solvent (A) = methanol and solvent (B) = 0.04% trifluoroacetic acid (TFA) in water. The total run time was 13 min and the mobile phase flow was as follows: (A)/(B) = 30/70 (0–13 min). The TAAR extract samples were analyzed using a UV detector at a wavelength of 327 nm.

### 2.4. Cell Culture

The HaCaT (human keratinocyte) cell line was purchased from the Korean Cell Line Bank (Seoul, Republic of Korea) and the RAW 264.7 (mouse macrophage) cell line was purchased from the American Type Culture Collection (ATCC, VA, USA) (ATCC; TIB-71). The cell lines were maintained in DMEM containing 10% heat-inactivated FBS and 1% of penicillin (100 units/mL)/streptomycin (100 μg/mL) (Welgene, Seoul, Republic of Korea). The cell lines were cultured in an incubator with a humidified atmosphere of 5% CO_2_ at 37 °C, and the medium was changed every 2 or 3 days during incubation.

### 2.5. Cell Viability Assay

The effect of TAAR extract on RAW 264.7 cell and HaCaT cell viability was assessed using the MTT assay. RAW 264.7 cells and HaCaT cells at a density of 1 × 10^4^ cells/well were seeded into a 96-well plate and treated with various concentrations of TAAR extract (0, 4, 8, 16, 25, 50, 100, and 200 μg/mL), and incubated for 24 h. Following incubation, 0.5 μg/mL final concentration of MTT solution was added to each well, and the plates were further incubated for 4 h in the dark. The supernatant of each well was then discarded, and crystallized formazan was dissolved in dimethyl sulfoxide (DMSO) (150 µL/well). The optical absorbance of the plates was measured at 570 nm wavelength using a Synergy HTX Multi-Mode microplate reader (BioTek, Winooski, VT, USA).

### 2.6. Real-Time Polymerase Chain Reaction (PCR)

RAW 264.7, and HaCaT cells were pre-treated with TAAR extract (0, 4, 8, and 16 μg/mL) for 2 h and treated with LPS (1 μg/mL) or TNF-α/IFN-γ (10 ng/mL) for 30 min. Total RNA from the cell lines was extracted using 1 mL TRIzol solution, and cDNA was synthesized using the Prime Super Script™ II cDNA synthesis kit (Takara Bio, Inc., Mountain View, CA, USA) according to the manufacturer’s instructions. Quantitative cDNA amplification was per-formed using the Applied Biosystems StepOne system (Applied Biosystems, Waltham, CA, USA) with the SYBR Green PCR Master Mix. Primer sequences are shown in Table 1. GAPDH was used to quantify relative mRNA expression.

### 2.7. Western Blotting Analysis

Protein expression of iNOS, COX-2, MAPKs, IκB kinase, NFκB, Nrf2, HO-1, NQO1, lamin B, and β-actin was detected by Western blotting. RAW 264.7 cells were pre-treated with various doses of TAAR extract (0, 4, 8, and 16 μg/mL) for 2 h, followed by treatment with LPS (1 μg/mL) for 30 min. HaCaT cells were pretreated with various doses of TAAR extract (0, 4, 8, and 16 μg/mL) for 2 h followed by treatment with TNF-α/IFN-γ (10 ng/mL) for 30 min. The cells were harvested and proteins were extracted using RIPA buffer containing protease and phosphatase inhibitors. The lysates were separated by 8–12 % SDS-PAGE and the protein bands were transferred onto PVDF membranes. After 2 h of blocking with 5% skim milk in Tris-buffered saline containing 0.1% Tween 20 (TBS-T) buffer, the membranes were incubated with primary antibodies overnight at 4 °C. The membranes were then washed twice with TBS-T buffer and immunoblotted with horseradish peroxidase (HRP)-conjugated secondary antibody for 1 h at room temperature. Finally, the membrane was exposed to an ECL solution kit using an image analyzer.

### 2.8. Animals

BALB/c mice (5 weeks, male, 21 ± 2 g) were provided by Samtako Bio Korea (Osan, Republic of Korea). All mice were housed in stainless steel mouse cages in a pathogen-free animal room with a controlled environment (temperature, 22 ± 1 °C; humidity, 55 ± 5%; 12 h light/12 h dark cycle). Mice were fed standard chow and tap water ad libitum. Animal experiments were performed in accordance with the guidelines of the Animal Experiment Ethics Committee of Jeonbuk National University (Confirmation No. JBNU 2022–06).

### 2.9. Induction of AD-like Skin Lesion in Mice

Before the experiment, all mice were acclimatized to the laboratory environment for 1 week. All mice were then randomly divided into the following six groups containing five mice each: (i) Normal group (control, vehicle treatment), (ii) DNCB group (treated with DNCB only), (iii) TAAR 40 group (treated with DNCB and oral administration of 40 mg/kg TAAR), (iv) TAAR 80 group (treated with DNCB and oral administration of 80 mg/kg TAAR), (v) TAAR 160 group (treated with DNCB and oral administration of 160 mg/kg TAAR) and (vi) Dexa group (positive control, treated with DNCB and oral administration of 1 mg/kg dexamethasone). DNCB was used to induce AD-like skin lesions in the dorsal skin of the mice. One day before the experiment, the dorsal skin hair of mice in all groups was removed. DNCB was dissolved in acetone and olive oil mixture (4:1, *v*/*v*) to prepare a 1% DNCB solution, and the dorsal skin of all mice except those in the normal group was treated with 200 μL of 1% DNCB once daily for 3 days. Subsequently, AD-like skin lesions were induced by treating the dorsal skin of mice with 200 μL of 0.5% DNCB solution once every 2 days for 10 days. The TAAR extract samples were dissolved in water and dexamethasone was dissolved in ethanol to make respective solution of each, and the TAAR (40 mg/kg, 80 mg/kg, and 160 mg/kg) and Dexa (1 mg/kg) groups were orally administered the relevant concentration daily for 11 days from day 4.

### 2.10. Measurement of AD Clinical Score, Dorsal Skin Thickness and Moisture Contents

On the last day of the experiment, the severity of AD symptoms (erythema, edema, and scratches) on the dorsal skin of all mice was visually assessed according to previously described criteria [25]. The dorsal skin was separated and mouse dorsal skin thickness was calculated as the average of three measurements using a micrometer. The moisture content (%) of the dorsal skin of each mouse was measured using a TS-skin diagnostic system (Aram Huvis Co., Seongnam, Republic of Korea) according to the manufacturer’s protocol.

### 2.11. Measurement of Serum IgE, IL-4, and Inflammatory Markers

The NO and PGE_2_ were measured in accordance with previous protocols [26]. Serum levels of IgE and IL-4 were measured using an ELISA assay kit (BioLegend, San Diego, CA, USA), according to the manufacturer’s instructions.

### 2.12. Histological Analysis

At the end of the experiment, the mice were sacrificed, and all dorsal skin tissue specimens obtained from mice were fixed in 10% formalin for 24 h at room temperature and then embedded in paraffin wax. The paraffin blocks were then serially cut into 4-µm-thick sections and mounted onto slides. The sections were stained with hematoxylin and eosin (H&E; hematoxylin for 1 min and eosin for 3 min) to determine histological changes in the skin and stained with toluidine blue (TB) to determine mast cell infiltration. Histological changes were analyzed using an optical microscope (Olympus CX21) and the sections were photographed.

### 2.13. Immunohistochemistry (IHC) Staining

IHC staining was conducted as previously described [26]. Briefly, all the slides were deparaffinized and rehydrated. Antigen retrieval was performed in 10 mM citrate buffer with 0.05% Tween 20 (pH 6.0) in the microwave for 10 min. Endogenous peroxidase activity of slide was blocked with 3% H_2_O_2_ solution (Dako) for 7 min and then pre-blocked using protein-blocking serum for 10 min. All slides were then incubated with specific anti-bodies against F4/80, Nrf2, HO-1, and NQO1 at 4 °C overnight. All slides were washed three times in TBS solution, and the following process was performed using a Universal Quick kit (Vectastain). All slides were stained with 3,3′-diaminobenzidine, counterstained for 10 min with hematoxylin, and mounted using a mounting aqueous solution.

### 2.14. Statistical Analysis

Statistical analysis of all data was performed using GraphPad Prism software (version 5.0; San Diego, CA, USA) and data were expressed as mean ± standard error of the mean (SEM) of at least three independent experiments. Differences between groups were analyzed using one-way analysis of variance (ANOVA), followed by Tukey’s post hoc test. Statistical significance was set at *p* < 0.05.

## 3. Results

### 3.1. HPLC Analysis of the TAAR Extract

**Figure 1:**
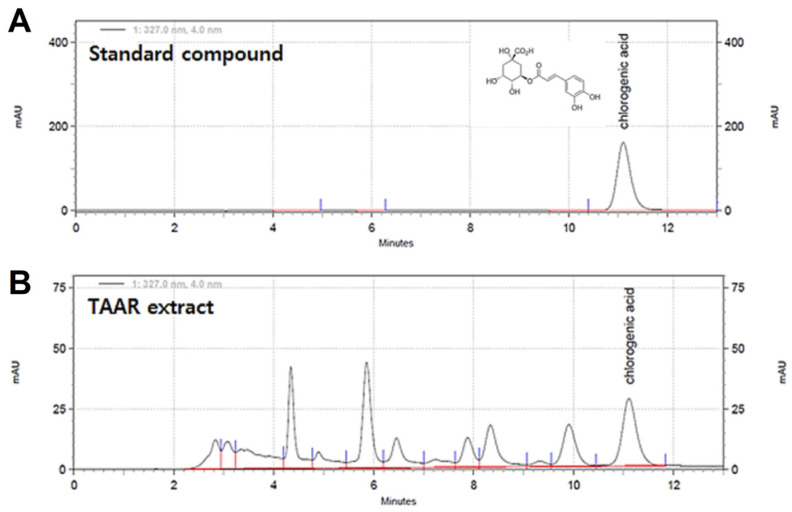
HPLC analysis of the TA and AR (TAAR) mixture extract. HPLC chromatograms of the (**A**) standard compound and (**B**) TAAR extract sample.

The main components of the TAAR extract were determined using HPLC with a PDA detector (Figure 1). HPLC revealed that one of the main components of the TAAR extract was chlorogenic acid, and the peak of chlorogenic acid in the TAAR extract was confirmed by comparison with the peak of the internal standard component. The concentration of chlorogenic acid in the TAAR extract was 0.297 mg/g. Chlorogenic acid is one of the well-known dietary polyphenols [27]. Chlorogenic acid is known to have important pharmacological and therapeutic functions such as anti-inflammatory, antioxidant, and antibacterial. Therefore, we anticipate that chlorogenic acid, a component contained in TAAR extract, is helpful in alleviating atopic dermatitis-like skin symptoms [28,29].

### 3.2. TAAR Extract Attenuates LPS-Induced Pro-Inflammatory Cytokines and Inflammatory Mediators in RAW 264.7 Cells

**Figure 2:**
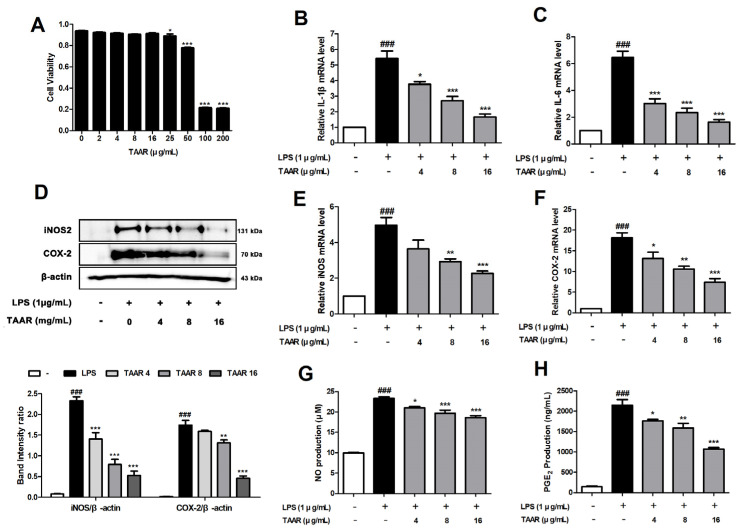
Effects of TAAR extract on the viability of and inflammatory response in LPS-induced RAW 264.7 cells. (**A**) Cytotoxicity of TAAR extract. mRNA levels of pro-inflammatory cytokines (**B**) IL-1β and (**C**) IL-6 in LPS-induced RAW 264.7 cells. Protein levels of (**D**) iNOS and COX-2 and the mRNA levels of (**E**) iNOS and (**F**) COX-2 in LPS-induced RAW 264.7 cells. Expression levels of (**G**) NO and (**H**) PGE_2_ in LPS-induced RAW 264.7 cells. All values are presented as the mean ± SEM of three experiments. Data were analyzed by Tukey’s post hoc test. ^###^ *p <* 0.001 vs. no treatment group; * p < 0.05, ** p < 0.01, and *** p < 0.001 vs. LPS treatment only group.

Macrophages that control the inflammatory response are characterized by the acute and chronic accumulation in inflamed lesions and play an important role in AD progression [30]. We first examined the effect of TAAR extract on the viability of RAW 264.7 cells using the MTT assay. We found that TAAR extract treatment at concentrations ranging from 0 to 16 μg/mL did not show cytotoxicity in RAW 264.7 cells (Figure 2A). Therefore, we selected these concentration ranges (4, 8, and 16 μg/mL) for further analysis. To deter-mine whether TAAR extract inhibited the expression of LPS-induced pro-inflammatory cytokines, we conducted real-time PCR. We found that the increased expression levels of cytokines (IL-1β and IL-6) in LPS-stimulated RAW 264.7 cells were suppressed by TAAR treatment in a concentration-dependent manner (Figure 2B,C). Next, the effect of TAAR extract on the expression levels of iNOS and COX-2 was measured using Western blotting and real-time PCR. The expression of iNOS and COX-2 protein and mRNA was significantly increased by LPS treatment and suppressed by TAAR treatment in a dose-dependent manner (Figure 2D–F). We also investigated the effect of TAAR extract on the expression of NO and PGE_2_, which are both LPS-induced inflammation mediators and downregulators of iNOS and COX-2. We found that TAAR treatment suppressed LPS-induced NO and PGE_2_ increased expression (Figure 2G,H).

### 3.3. TAAR Extract Suppresses the Expression of TNF-α/IFN-γ-Induced Pro-Inflammatory Cytokines and Chemokines in HaCaT Cells

**Figure 3:**
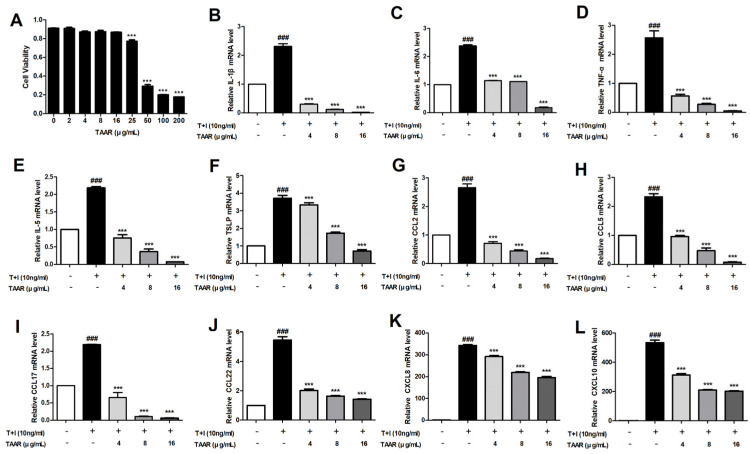
Effects of TAAR extract on the viability of and the inflammatory response in TNF-α/IFN-γ-induced HaCaT cells. (**A**) Cytotoxicity of TAAR extract. mRNA expression of (**B**–**F**) cytokines IL-1β, IL-6, TNF-α, IL-5, and TSLP and (**G**–**L**) chemokines CCL2, CCL5, CCL17, CCL22, CXCL8, CXCL10. All values are presented as the mean ± SEM of three experiments. Data were analyzed by Tukey’s post hoc test. ^###^ *p* <0.001 vs. no treatment group; *** p < 0.001 vs. TNF-α/IFN-γ treatment only group.

Skin immune diseases such as AD and allergies are caused by excessive skin inflammation. As keratinocytes activated by various stimuli play an important role in the inflammatory immune response, we examined the anti-inflammatory effects of TAAR ex-tract on HaCaT cells under TNF-α/IFN-γ-induced inflammatory conditions [31]. First, we investigated the viability of HaCaT cells treated with TAAR extract. Similar to RAW 264.7 cells, TAAR extract did not show significant cytotoxicity in HaCaT cells at concentrations of 0 to 16 μg/mL (Figure 3A). Therefore, subsequent experiments were performed at TAAR concentrations of 4, 8, and 16 μg/mL. The effects of TAAR extract on the expression of TNF-α/IFN-γ-induced pro-inflammatory cytokines and chemokines in HaCaT cells were assessed using real-time PCR. We found that TNF-α/IFN-γ significantly increased the mRNA expression levels of pro-inflammatory cytokines (IL-1β, IL-6, TNF-α, IL-5, and TSLP), whereas TAAR extract significantly decreased the expression of inflammatory cytokines in HaCaT cells (Figure 3B–F). We then assessed the effect of TAAR extract on TNF-α/IFN-γ-induced chemokine production that acts as inflammatory mediators in HaCaT cells. We found that TAAR extract effectively suppressed TNF-α/IFN-γ-induced production of chemokines (CCL2 (MCP-1), CCL5 (RANTES), CCL17 (TARC), CCL22 (MDC), CXCL8 (IL-8), and CXCL10) in HaCaT cells (Figure 3G–L).

### 3.4. TAAR Extract Inhibits Inflammation through the Regulation of NFκB and MAPKs Signaling Pathway in TNF-α/IFN-γ-Induced HaCaT Cells

**Figure 4:**
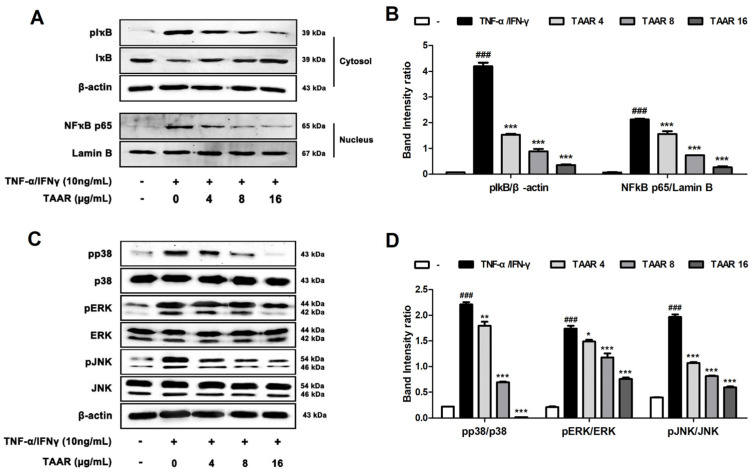
Effects of TAAR extract on the NFκB and MAPK signaling pathways in TNF-α/IFN-γ-induced HaCaT cells. (**A**) Protein levels of cytosol IκB and nucleus NFκB. (**B**) Bar graph showing the relative density of the Western blot band pIκB/β-actin and NFκB/lamin B. (**C**) Protein expression levels of phosphorylated and total MAPKs (p38, ERK, and JNK). (**D**) Bar graph showing the relative density of the Western blot band pp38/p38, pERK/ERK, and pJNK/JNK. All values are presented as the mean ± SEM of three experiments. Data were analyzed by Tukey’s post hoc test. ^###^ *p* < 0.001 vs. no treatment group; * p < 0.05, ** p < 0.01, and *** p < 0.001 vs. TNF-α/IFN-γ treatment only group.

To evaluate the anti-inflammatory mechanism of TAAR extract, we assessed the expression of proteins of the NFκB and MAPK signaling pathways, which are major inflammatory mediators of AD, by Western blotting. As shown in Figure 4A,B, TNF-α/IFN-γ stimulation increased the phosphorylation of IkB in the cytosol and the expression of NFκB in the nucleus. However, treatment with TAAR extract significantly downregulated the phosphorylation of IkB and the expression of NFκB. This suggests that the TAAR extract effectively inhibits the translocation of p65 from the cytosol to the nucleus in TNF-α/IFN-γ-induced HaCaT cells. In addition, TNF-α/IFN-γ stimulation in-creased the phosphorylation of p38, ERK, and JNK in HaCaT cells. However, in HaCaT cells treated with TAAR extract, the phosphorylation of all proteins of the MAPK signaling pathway decreased considerably in a concentration-dependent manner (Figure 4C,D). These results indicated that TAAR extract suppressed the expression of pro-inflammatory cytokines and chemokines by blocking the activation of the NFκB and MAPK signaling pathways.

### 3.5. TAAR Extract Activates Nrf2/HO-1/NQO1 Signaling Pathway in TNF-α/IFN-γ-Induced HaCaT Cells

**Figure 5:**
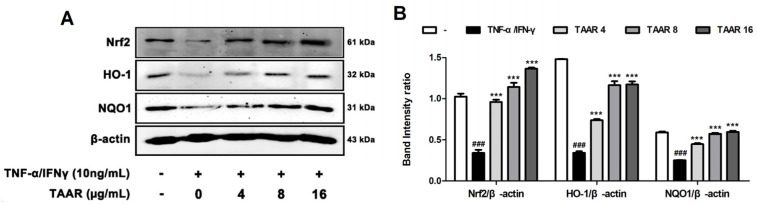
Effects of TAAR extract on the activation of the Nrf2/HO-1/NQO1 signaling pathway in TNF-α/IFN-γ-induced HaCaT cells. (**A**) Protein levels of Nrf2, HO-1, and NQO1. (**B**) Bar graph showing the relative density of the Western blot band Nrf2/β-actin, HO-1/β-actin, and NQO1/β-actin. All values are presented as the mean ± SEM of three experiments. Data were analyzed by Tukey’s post hoc test. ^###^ *p* < 0.001 vs. no treatment group; *** p < 0.001 vs. TNF-α/IFN-γ treatment only group.

To examine the mechanisms by which TAAR extract manifests its antioxidant effects against TNF-α/IFN-γ-induced oxidative stress, we assessed the protein expression of various oxidative stress-related factors, such as Nrf2, HO-1, and NQO1, using Western blotting. We found that the reduced expression of Nrf2, HO-1, and NQO1 in HaCaT cells stimulated with TNF-α/IFN-γ was gradually increased by treatment with TAAR extract (Figure 5A,B). These results suggest that treatment with TAAR extract ameliorates oxidative stress in TNF-α/IFN-γ-induced HaCaT cells through the increased expression of Nrf2, HO-1, and NQO1.

### 3.6. TAAR Extract Alleviates AD-like Symptoms in DNCB-Induced Mice

**Figure 6:**
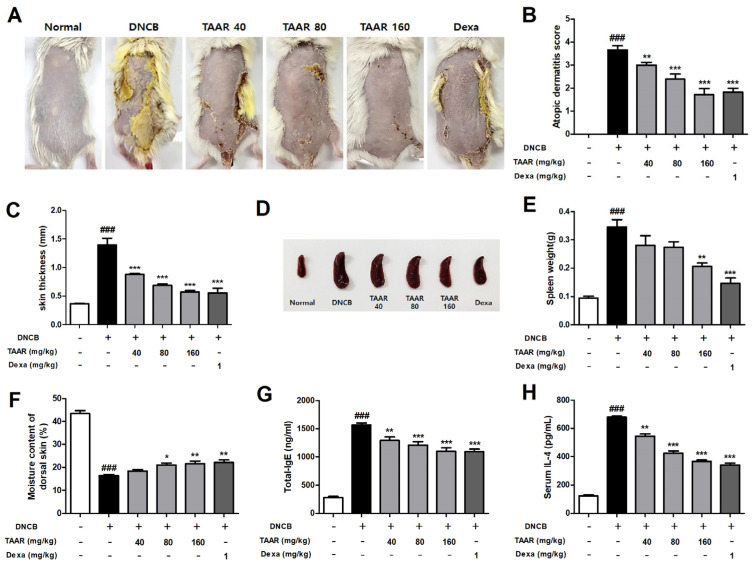
Effect of TAAR extract on DNCB-induced AD-like clinical symptoms in mice. (**A**) Representative photographic images of AD-like dorsal skin lesions from mice from each group. (**B**) Atopic dermatitis score and (**C**) skin thickness. (**D**) Representative photographs of mouse spleen and (**E**) spleen weight of mice from each group. (**F**) Moisture content of dorsal skin. (**G**) Total serum IgE and (**H**) serum IL-4 levels. All values are presented as the mean ± SEM of three experiments. Data were analyzed by Tukey’s post hoc test. ^###^ *p* < 0.001 vs. normal control group; * p < 0.05, ** p < 0.01, and *** p < 0.001 vs. DNCB-treated only group.

DNCB was repeatedly applied to the dorsal skin of mice to induce AD-like skin lesions. Oral administration of TAAR extract and dexamethasone daily for 11 days in DNCB-induced mice significantly improved AD-like dorsal skin lesions (Figure 6A). In addition, we evaluated the AD severity score of mouse dorsal skin lesions by referring to known criteria. As shown in Figure 6B, the severity score was significantly reduced in the TAAR extract treated group in a dose-dependent manner. Dorsal skin thickness was measured after sacrificing all mice. The increased thickness of the dorsal skin in mice by DNCB was reduced by oral administration of TAAR extract in a dose-dependent manner (Figure 6C). The spleen is one of the most important lymphoid organs and is enlarged during an inflammatory response. First, for visual comparison of the spleen, the spleen representing the average weight of each group was selected and photographed (Figure 6D). The spleen index (spleen weight/body weight (g)) of each representative spleen was computed. The spleen index increased by DNCB and was recovered by TAAR extract treatment (Figure 6E). AD is characterized by skin barrier function damage due to loss of skin moisture; therefore so we investigated the effect of TAAR extract on transepidermal water loss in mice dorsal skin [32]. As shown in Figure 6F, DNCB-treated mice showed markedly reduced dorsal skin moisture content (%) compared to the normal control group. Furthermore, compared to the DNCB-treated group, the skin moisture content (%) was re-stored by oral administration of the TAAR extract. IgE and IL-4 are known to play crucial roles in the development and progression of AD and are associated with the T helper 2 (Th2)-related AD immune response [33]. Therefore, we measured the serum levels of total IgE and IL-4 in mice using ELISA. As shown in Figure 6G and H, total IgE and IL-4 levels were significantly elevated by DNCB treatment but were suppressed by oral administration of TAAR extract. Taken together, these results indicate that TAAR extract exhibits an admirable effect in relieving many AD-related clinical symptoms.

### 3.7. TAAR Extract Decreases Epidermal Thickness and Mast Cell Infiltration in Mice with DNCB-Induced AD-like Skin Lesions

**Figure 7:**
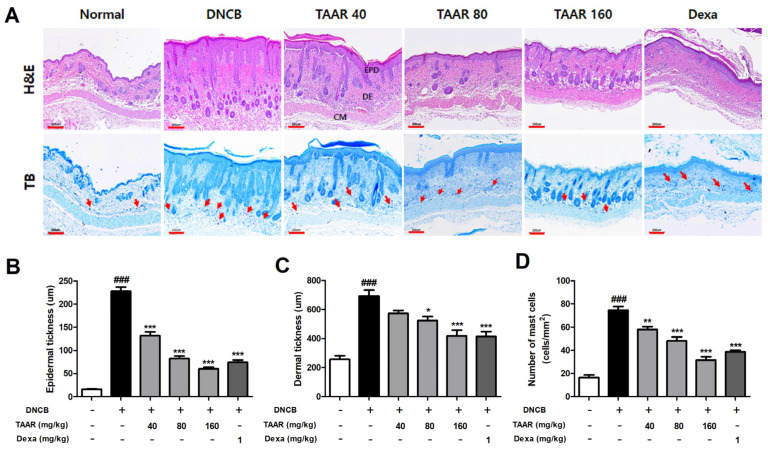
Effects of TAAR extract on histological features in the dorsal tissue of DNCB-induced mice. (**A**) Representative images of H&E and TB staining of the mice dorsal skin tissue (scale bar: 200 µm). Mast cells are indicated by red arrows. (**B**) Epidermal thickness, (**C**) dermal thickness, and (**D**) number of infiltrated mast cells. All values are presented as the mean ± SEM of three experiments. Data were analyzed by Tukey’s post hoc test. ^###^ *p* < 0.001 vs. normal control group; * p < 0.05, ** p < 0.01, and *** p < 0.001 vs. DNCB-treated only group.

We performed H&E and TB staining, respectively, to observe DNCB-induced dorsal skin epidermal hyperkeratosis, hypertrophy, and mast cell infiltration (Figure 7A). H&E staining revealed that epidermal and dermal thicknesses were significantly greater in the DNCB group than in the normal control group, but treatment with TAAR extract reduced DNCB-induced epidermal and dermal thicknesses (Figure 7B,C). The number of infiltrated mast cells in the dermis stained with TB was significantly higher in the DNCB group than in the normal group. However, oral administration of TAAR extract decreased mast cell infiltration in a dose-dependent manner (Figure 7D and Figure S1). In addition, TAAR extract alleviated macrophages infiltration (F4/80) in the mouse dorsal tissue in a dose-dependent manner in DNCB-induced mice. High-dose TAAR extract treatment was similarly effective to dexamethasone for attenuating macrophages infiltration (Figure S2).

### 3.8. TAAR Extract Reduces the Expression of DNCB-Induced Pro-Inflammatory Cytokines and Chemokines in Mice

**Figure 8:**
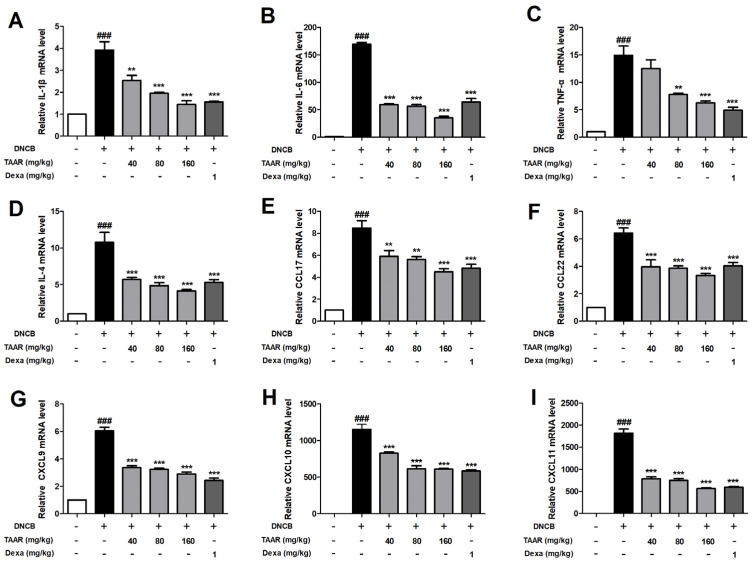
Effects of TAAR extract on the mRNA levels of DNCB-induced pro-inflammatory cytokines and chemokines in the dorsal skin tissue of DNCB-induced mice. mRNA expression of (**A**–**D**) cytokines IL-1β, IL-6, TNF-α, and IL-5 and (**E**–**I**) chemokines CCL17, CCL22, CXCL8, CXCL10, CXCL11. All values are presented as the mean ± SEM of three experiments. Data were analyzed by Tukey’s post hoc test. ^###^ *p* < 0.001 vs. normal control group; ** p < 0.01, and *** p < 0.001 vs. DNCB-treated only group.

Real-time PCR was used to assess the inhibitory effect of the TAAR extract on the expression of pro-inflammatory cytokines and chemokines in the dorsal skin tissue of DNCB-induced AD-like mice. As indicated in Figure 8, DNCB treatment significantly in-creased the production of pro-inflammatory cytokines (IL-1β, IL-6, TNF-α, and IL-4) and chemokines (CCL17 (TARC), CCL22 (MDC), CXCL9, CXCL10, and CXCL11) compared to the normal control group. However, DNCB-induced pro-inflammatory cytokine and chemokine production was markedly reduced by oral administration of TAAR extract. Therefore, TAAR extract effectively decreased inflammation by suppressing the expression of pro-inflammatory cytokines and chemokines in mice with AD-like skin lesions.

### 3.9. TAAR Extract Promoted Nrf2/HO-1/NQO1 Pathway Activation in Mice with DNCB-Induced AD-like Skin Lesions

**Figure 9:**
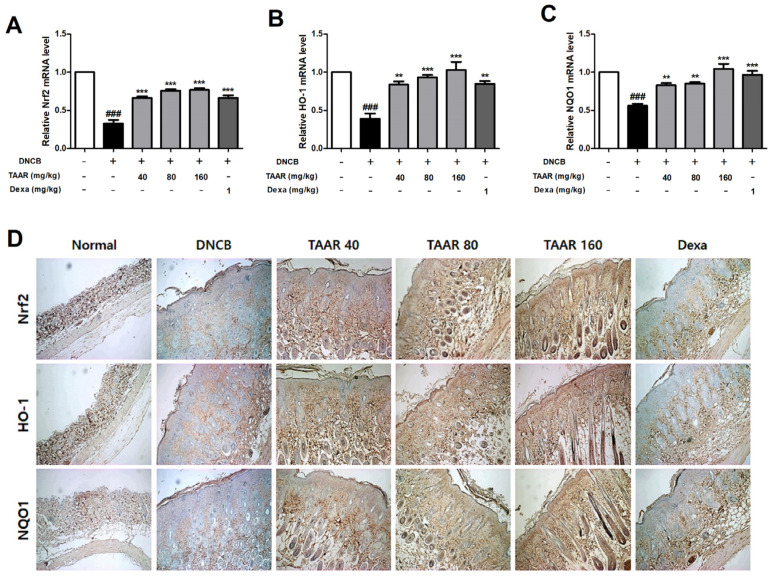
Effects of TAAR extract on the levels of oxidative stress-related factors in the dorsal skin tissue of DNCB-induced mice. (**A**–**C**) mRNA levels of Nrf2, HO-1, and NQO1 in the dorsal skin tissue of DNCB-induced mice. (**D**) Protein expression of Nrf2, HO-1, and NQO1 in dorsal skin tissue of DNCB-induced mice. (100× magnification). All values are presented as the mean ± SEM of three experiments. Data were analyzed by Tukey’s post hoc test. ^###^ *p* < 0.001 vs. normal control group; ** p < 0.01, and *** p < 0.001 vs. DNCB-treated only group.

Real-time PCR and IHC staining were performed to investigate the expression of oxidative stress-related pathways (Nrf2/HO-1/NQO1 signaling pathway) in the dorsal skin tissue of mice. As shown in Figure 9A–C, the mRNA expression levels of Nrf2, HO-1, and NQO1 in the DNCB group were lower than those in the control group but were considerably higher in the TAAR extract-treated groups than in the DNCB group. In line with these results, the DNCB-induced decreased protein levels of Nrf2, HO-1, and NQO1 were elevated by the oral administration of TAAR extract. In particular, according to the results of IHC staining, the Dexa group showed almost non-existent of antioxidant effect compared to the TAAR extract-treated groups (Figure 9D and Figure S3).

## 4. Discussion

AD is a chronic relapsing inflammatory skin disease characterized by pruritus, eczema, erythema, and edema and is widely prevalent worldwide. Recently, inflammatory responses and oxidative stress have been shown to be involved in the pathogenesis and progression of AD [34]. Drugs such as corticosteroids, calcineurin inhibitors, and antihistamines, currently known to treat AD, cause serious side effects with long-term use. Therefore, there is a need to develop natural plant-derived drugs with good efficacy and minimal adverse effects for the treatment of AD.

AD is closely related to keratinocytes and immune cells. Therefore, we assessed the anti-inflammatory and antioxidant effects and the underlying molecular mechanism of TAAR extract in mouse RAW 264.7 macrophages and human keratinocytes HaCaT. In addition, the anti-AD and immunomodulatory effects of TAAR extract were investigated in a mouse model of DNCB-induced AD-like skin lesions.

NO and PGE_2_, downstream signaling factors of iNOS and COX-2, and various other pro-inflammatory cytokines and chemokines are involved in the regulation of immune and inflammatory responses and cause symptoms such as pain, fever, and edema [35]. Therefore, we first evaluated the anti-inflammatory effects of the TAAR extract by measuring the levels of inflammation-related factors in RAW 264.7 and HaCaT cells. We found that TAAR extract reduced the mRNA expression of LPS-induced increased levels of pro-inflammatory cytokines IL-1β and IL-6 in RAW 264.7 macrophages. In addition, the LPS-induced increase in iNOS, COX-2, NO, and PGE_2_ was decreased by pretreatment with TAAR extract in a dose-dependent manner.

Similar to the results in mouse macrophage RAW 264.7, the anti-inflammatory effect of TAAR extract was also observed in human keratinocyte HaCaT cells in controlling the inflammatory environment induced by TNF-α/IFN-γ.

Keratinocytes are important in the formation of the stratum corneum and act as targets of the immune response in the skin. When the epidermal barrier of the skin is damaged by physical or chemical stimuli, many cytokines (IL-1β, IL-6, TNF-α, IL-5, and TSLP) and chemokines (MCP-1, RNATES, TARC, MDC, CXCL8, and CXCL10) are expressed in stimulated keratinocytes to promote the progression of inflammatory skin diseases [36]. Therefore, inhibition of the secretion of pro-inflammatory cytokines and chemokines may be a potential therapeutic strategy for AD.

Increased activation of the NFκB and MAPK signaling pathways in the skin in AD triggers the production of various cytokines and chemokines that mediate the inflammatory response [37]. Indeed, in keratinocytes, the activation of the NFκB and MAPK signaling pathways is induced by TNF-α/IFN-γ and modulates various downstream inflammation-related factors. Therefore, to study the anti-inflammatory effects and related mechanisms of TAAR extract in keratinocytes, we investigated the activation of NFκB and MAPK signaling pathways. We found that TAAR extract suppressed the mRNA levels of various pro-inflammatory cytokines and chemokines in TNF-α/IFN-γ-induced HaCaT cells. In addition, TAAR extract reduced the activation of the NFκB and MAPK signaling pathways and suppressed the degradation of IκB. Based on these results, we postulate that TAAR extract exerts a protective effect against skin inflammation by inhibiting the production of pro-inflammatory cytokines and chemokines by suppressing the activation of the NFκB and MAPK signaling pathways in inflammatory skin conditions such as AD.

Excessive damage to the antioxidant defense system of the skin exacerbates AD progression. The secretion of cytokines by the inflammatory response promotes the production of reactive oxygen species in immune cells, causing oxidative stress. The continuous release of reactive oxygen species and accumulation of oxidative stress are related to the weakening of the immune system and increased susceptibility to various diseases such as AD [38]. The increase in reactive oxygen species induces the migration of inflammatory cells, causing damage to skin tissue. Nrf2, HO-1, and NQO1 are antioxidant-related proteins that are involved in protecting cells from oxidative stress and inflammatory responses. Under oxidative stress, the antioxidant-associated transcription factor Nrf2 moves from the cytosol to the nucleus where it promotes the production of antioxidant enzyme genes such as HO-1 and NQO1, leading to cell protection [39]. In addition, the Nrf2/HO-1/NQO1 signaling pathway is involved in the inflammatory response and attracts inflammatory cells to inflammatory lesions [40]. Therefore, targeting the activation of Nrf2/HO-1/NQO1 signaling could be an important treatment strategy for AD. In this study, we found that the expression levels of Nrf2, HO-1, and NQO1 were decreased in TNF-α/IFN-γ-induced HaCaT cells by inhibiting the antioxidant signaling pathway. However, treatment with TAAR extract increased the expression levels of Nrf2, HO-1, and NQO1, thus activating the antioxidant system.

The in vivo study was performed by repeatedly treating BALB/c mice with DNCB to induce AD-like skin lesions. TAAR extract restored AD-like skin lesions in DNCB-induced mice and significantly restored the severity of AD score. In addition, TAAR extract alleviated DNCB-induced thickened skin and transepidermal water loss and ameliorated the spleen index.

Inflammatory skin diseases such as AD are characterized by the overproduction of Th2-related cytokines. IL-4 is known to induce pruritus by inducing the synthesis of IgE and activating mast cells, which, in turn, contributes to the progression of AD [41]. We evaluated the inhibitory effects of TAAR extract on IgE, pro-inflammatory cytokines, and chemokines in DNCB-induced AD-like skin lesions in mice. Treatment with TAAR extract decreased serum total IgE and IL-4 levels in DNCB-induced mice. In addition, the DNCB-induced increase in pro-inflammatory cytokine and chemokine expression levels was reduced by TAAR extract treatment. Histopathological analysis showed that treatment with TAAR extract suppressed DNCB-induced epidermal and dermal thickening and mast cell infiltration.

**Figure 10:**
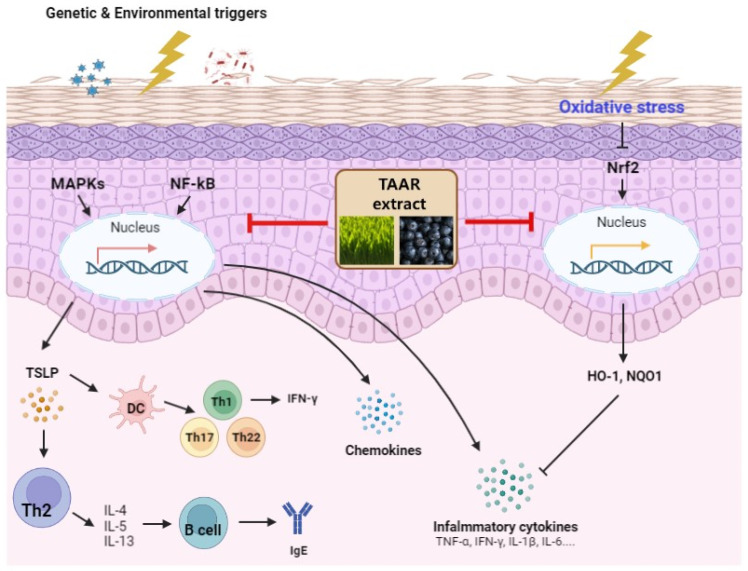
A pathomechanism summary of the anti-inflammatory and antioxidant effects of the TAAR extract.

We confirmed the activation of the Nrf2/HO-1/NQO1 signaling pathway to investigate the antioxidant effect of TAAR extract. Our results indicated that TAAR extract protected mice by restoring the expression of Nrf2, HO-1, and NQO1, which was downregulated by DNCB. Therefore, our results indicate that TAAR extract improves the DNCB-induced AD inflammatory response by activating the Nrf2/HO-1/NQO1 signaling pathway to inhibit the release of oxidative stress and inflammatory mediators (Figure 10).

## 5. Conclusions

We investigated the anti-inflammatory and antioxidative effects of the TAAR extract in vitro and in vivo. In vitro results showed that TAAR extract has an anti-inflammatory effect on LPS-induced macrophages and anti-inflammatory and antioxidative effects on TNF-α/IFN-γ-induced keratinocytes. In vivo results showed that TAAR extract has anti-inflammatory, antioxidative, and anti-AD effects on DNCB-induced AD-like skin lesions in mice. Therefore, our findings demonstrate that TAAR extract, with anti-inflammatory and antioxidant effects in inflammatory skin diseases such as AD, should be considered a potential natural therapeutic candidate for AD.

## Figures and Tables

**Table 1:** Primer sequences for real-time PCR.

Gene	Gene ID	Forward	Reverse
hTNF-α	7124	TTGGAGTGATCGGCCCCCAG	ACAGGCTTGTCACTCGGGGTT
hIL-1β	3553	CAGCTCTCTCCTTTCAGGGCCA	GGCCGTGGTTTCTGTCAGGC
hIL-5	3567	GCTAGCTCTTGGAGCTGCCT	CTTCAGTGCACAGTTGGTGA
hIL-6	3569	CTCCACAAGCGCCTTCGGTC	TGTGTGGGGCGGCTACATCT
hTSLP	85480	GCCATGAAAACTAAGGCTGC	CGCCACAATCCTTGTAATTG
hCCL2	6347	CAA ACTGAAGCTCGCACTC	CATTTCCACAATAATATTTTAG
hCCL5	6352	CGCTGTCATCCTCATTGCTA	GCACTTGCCACTGGTGTAGA
hCCL17	6361	CCATTCCCCTTAGAA AGCTG	CTCTCAAGGCTTTGCAGGTA
hCCL22	6367	TGCCGTGATTACGTCCGTTAC	AAGGCCACGGTCATCAGAGTAG
hCXCL8	3576	ACCGGAGCACTCCATAAGGCA	AGGCTGCCAAGAGAGCCACG
hCXCL10	3627	TTGCTGCCTTATCTTTCTGACTC	ATGGCCTTCGATTCTGGATT
miNOS	18126	CAGCTGGGCTGTACAAAC	CATTGGAAGTGAAGCGATTCG
mCOX-2	5912281	GAAGTCTTTGGTCTGGTGCCTG	GTCTGCTGGTTTGGAATAGTTGC
mTNF-α	21926	TAGCCAGGAGGGAGAACAGA	TTTTCTGGAGGGAGATGTGG
mIL-1β	16176	CTCCATGAGCTTTGTACAAGG	TGCTGATGTACCAGTTGGGG
mIL-4	16189	ATGGGTCTCAACCCCCAGCTA	TGCATGGCGTCCCTTCTCCT
mIL-6	16193	GACAACCACGGCCTTCCCTA	GGTACTCCAGAAGACCAGAGGA
mCCL17	20295	GGATGCCATCGTGTTTCTGA	GCCTTCTTCACATGTTTGTCTTTG
mCCL22	20299	ATTCTGGGAGTTTCAGGC	ATTCTGAGCCTGCTCCTT
mCXCL9	17329	GCAGTGTGGAGTTCGAGGAA	TCTAGGCAGGTTTGATCTCC
mCXCL10	15945	CTGAGTGGGACTCAAGGGA	TCGTGGCAATGATCTCAACAC
mCXCL11	56066	GGCAGAGATCGAGAAAGCT	ATTGCCTGCATTATGAGGCG
mNrf2	18024	ACCAAGGGGCACCATATAAAAG	CTTCGCCGAGTTGCACTC
mHO-1	15368	CAGAACCAGCCTGAACTAGC	TGGATGTGTACCTCCTTGGT
mNQO1	18104	ACAGGTGAGCTGAAGGACTC	GTTGTCGTACATGGCAGCAT
GAPDH	14433	CATGGCCTTCCGTGTTC	CCTGGTCCTCAGTGTAGC

## Data Availability

The data are contained within the article and Supplementary Materials.

## Supplementary Materials

The following supporting information can be downloaded at: https://www.mdpi.com/article/10.3390/antiox12010027/s1, Figure S1: Effects of TAAR extract on histological features in the dorsal tissue of DNCB-induced mice; Figure S2: Effects of TAAR extract on the levels of macrophage infiltration in the dorsal skin tissue of DNCB-induced mice; Figure S3: Effects of TAAR extract on the levels of oxidative stress-related factors in the dorsal skin tissue of DNCB-induced mice.
