# Supplementary material for: Extract of Wheatgrass and Aronia Mixture Ameliorates Atopic Dermatitis-Related Symptoms by Suppressing Inflammatory Response and Oxidative Stress In Vitro and In Vivo
Source: Antioxidants (Basel). 2022 Dec 23;12(1):27. doi: 10.3390/antiox12010027 (PMC9854678; doi:10.3390/antiox12010027)

Supplementary Figure S1. Effects of TAAR extract on histological features in the dorsal tissue of DNCB-induced mice. Representative images of TB staining of the mice dorsal skin tissue (scale bar: 200 μm).

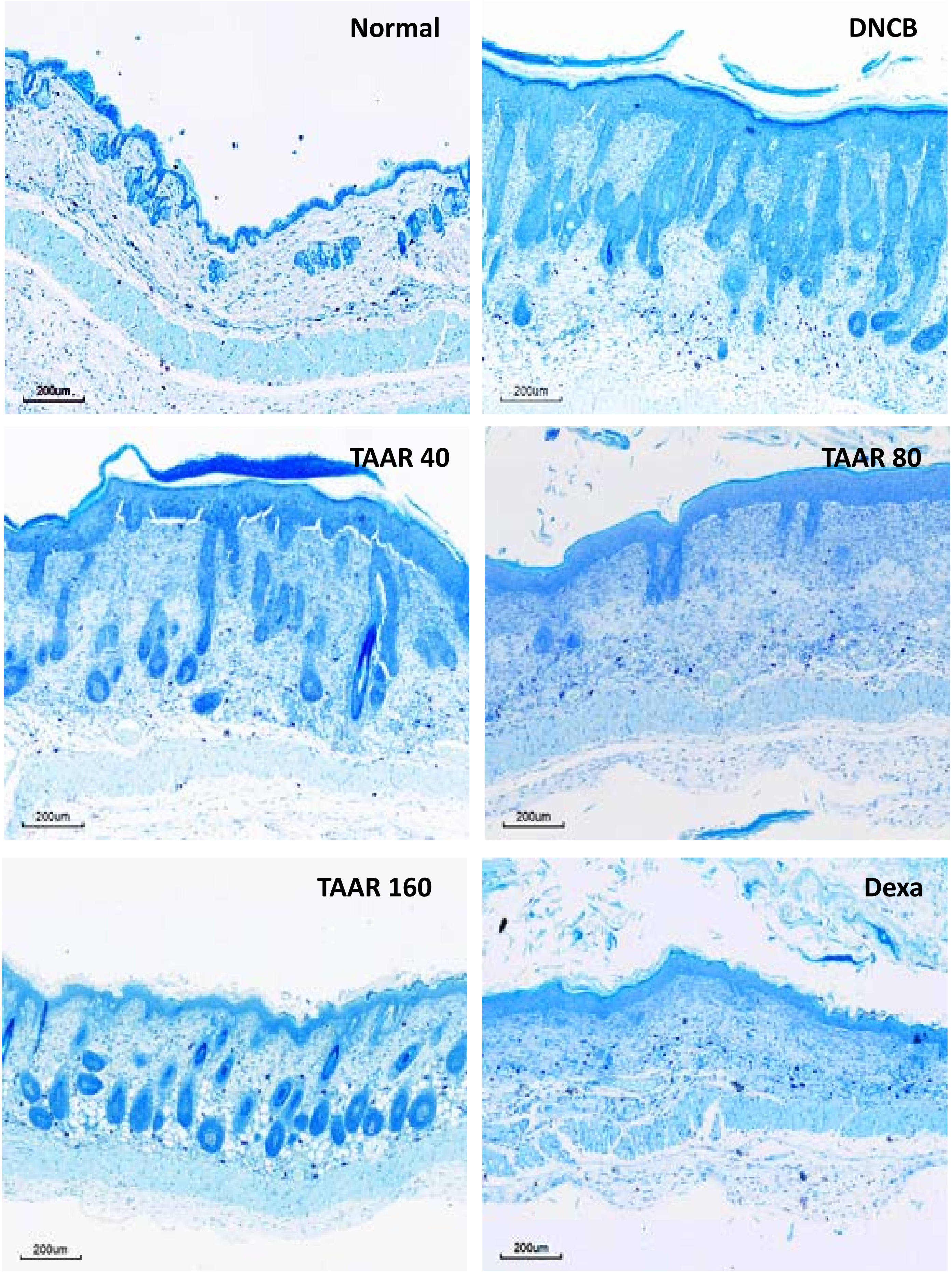

Supplementary Figure S2. Effects of TAAR extract on the levels of macrophage infiltration in the dorsal skin tissue of DNCB-induced mice. Protein expression of F4/80 in dorsal skin tissue of DNCB-induced mice.

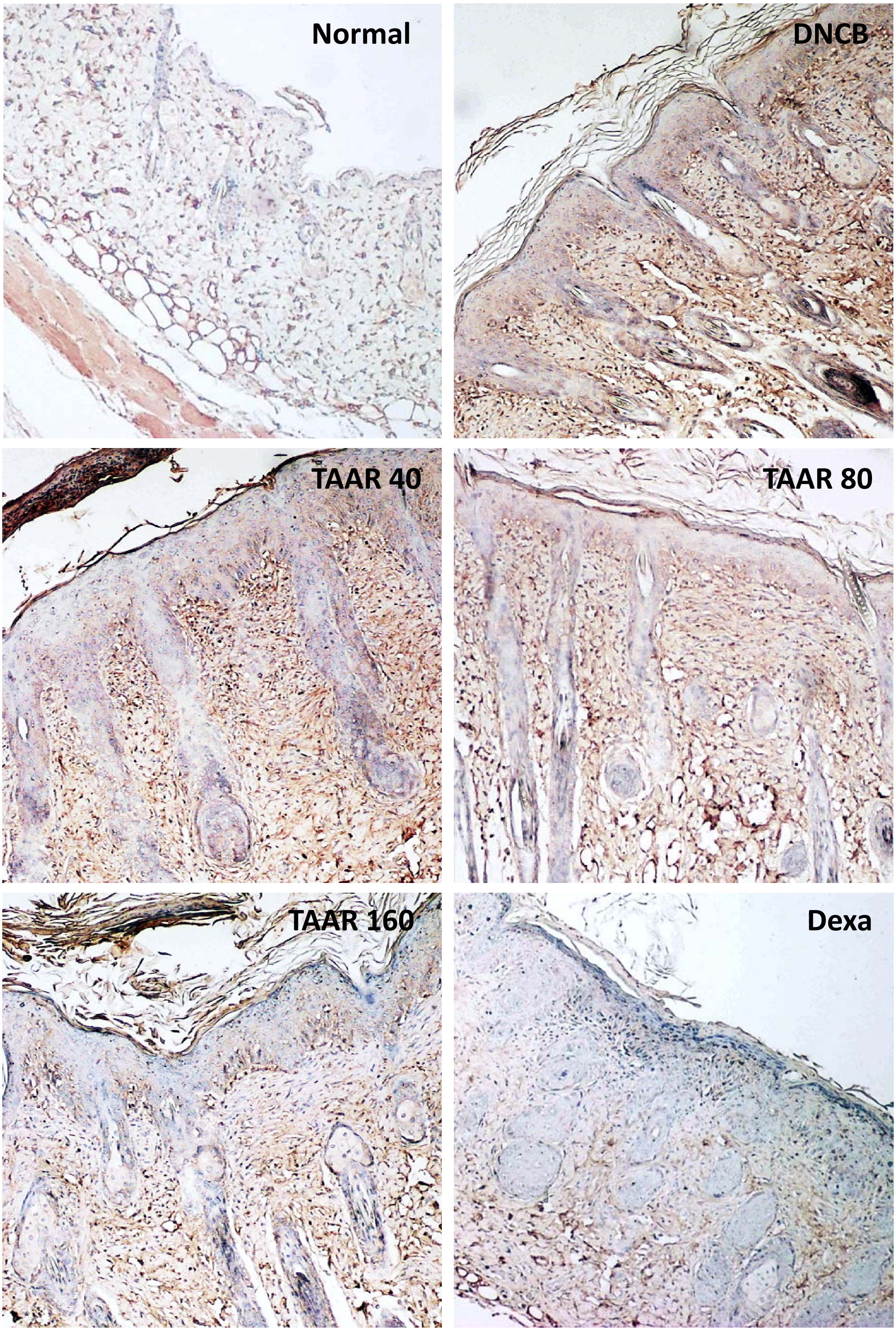

Supplementary Figure S3. Effects of TAAR extract on the levels of oxidative stress-related factors in the dorsal skin tissue of DNCB-induced mice. Protein expression of Nrf2 in dorsal skin tissue of DNCB-induced mice. (100× magnification).

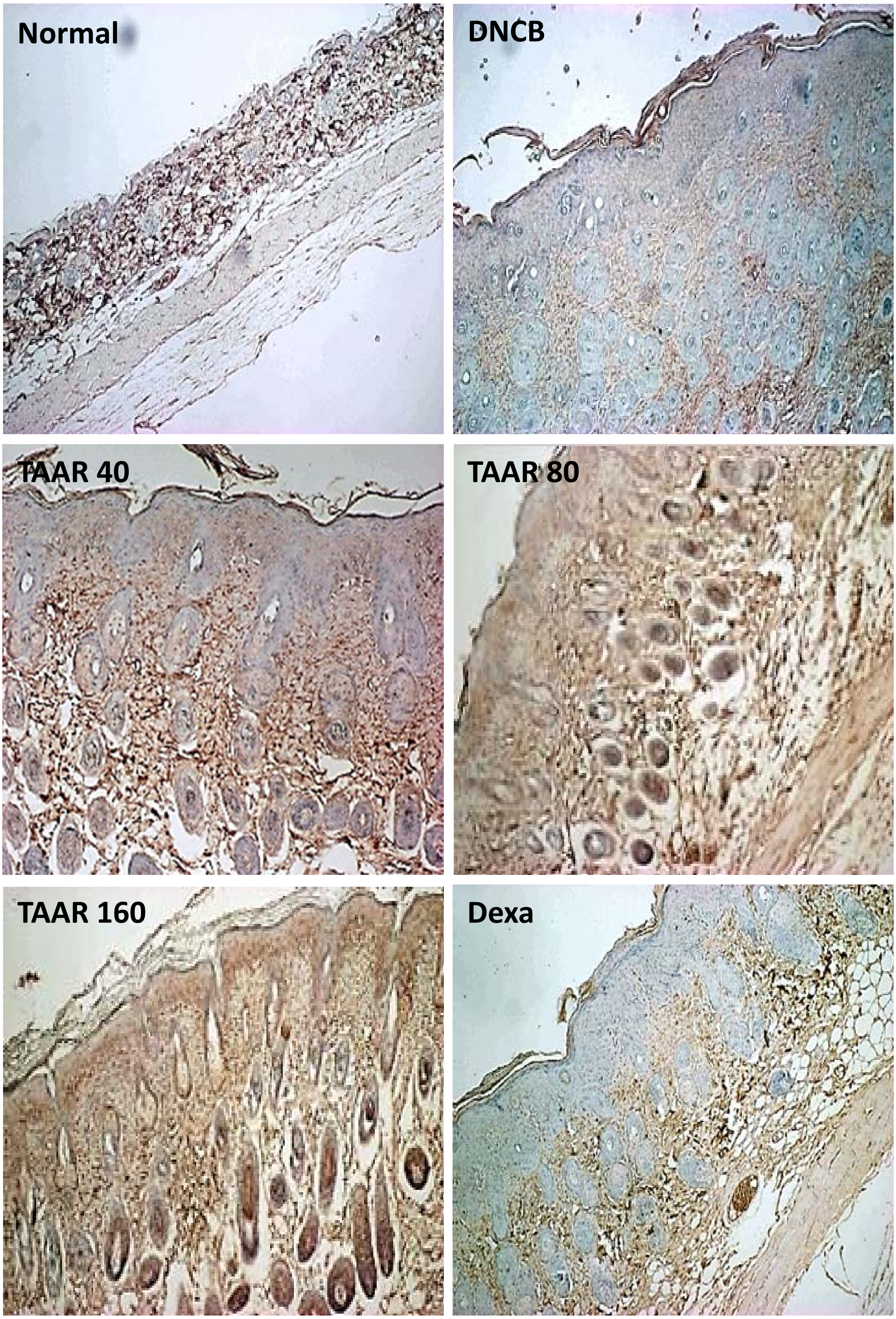

Supplementary Figure S3. Effects of TAAR extract on the levels of oxidative stress-related factors in the dorsal skin tissue of DNCB-induced mice. Protein expression of HO-1 in dorsal skin tissue of DNCB-induced mice. (100× magnification).

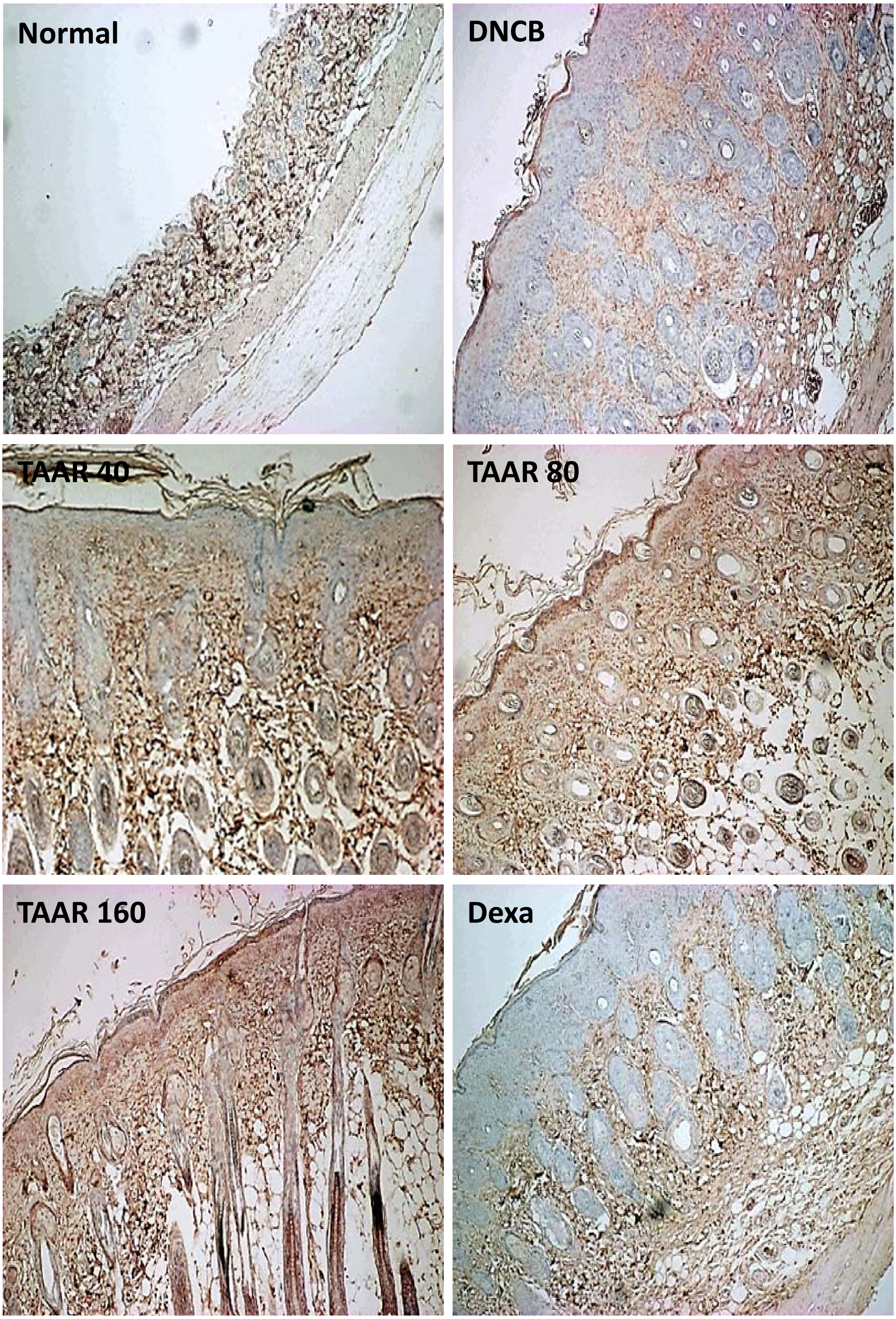

Supplementary Figure S3. Effects of TAAR extract on the levels of oxidative stress-related factors in the dorsal skin tissue of DNCB-induced mice. Protein expression of NQO1 in dorsal skin tissue of DNCB-induced mice. (100× magnification).

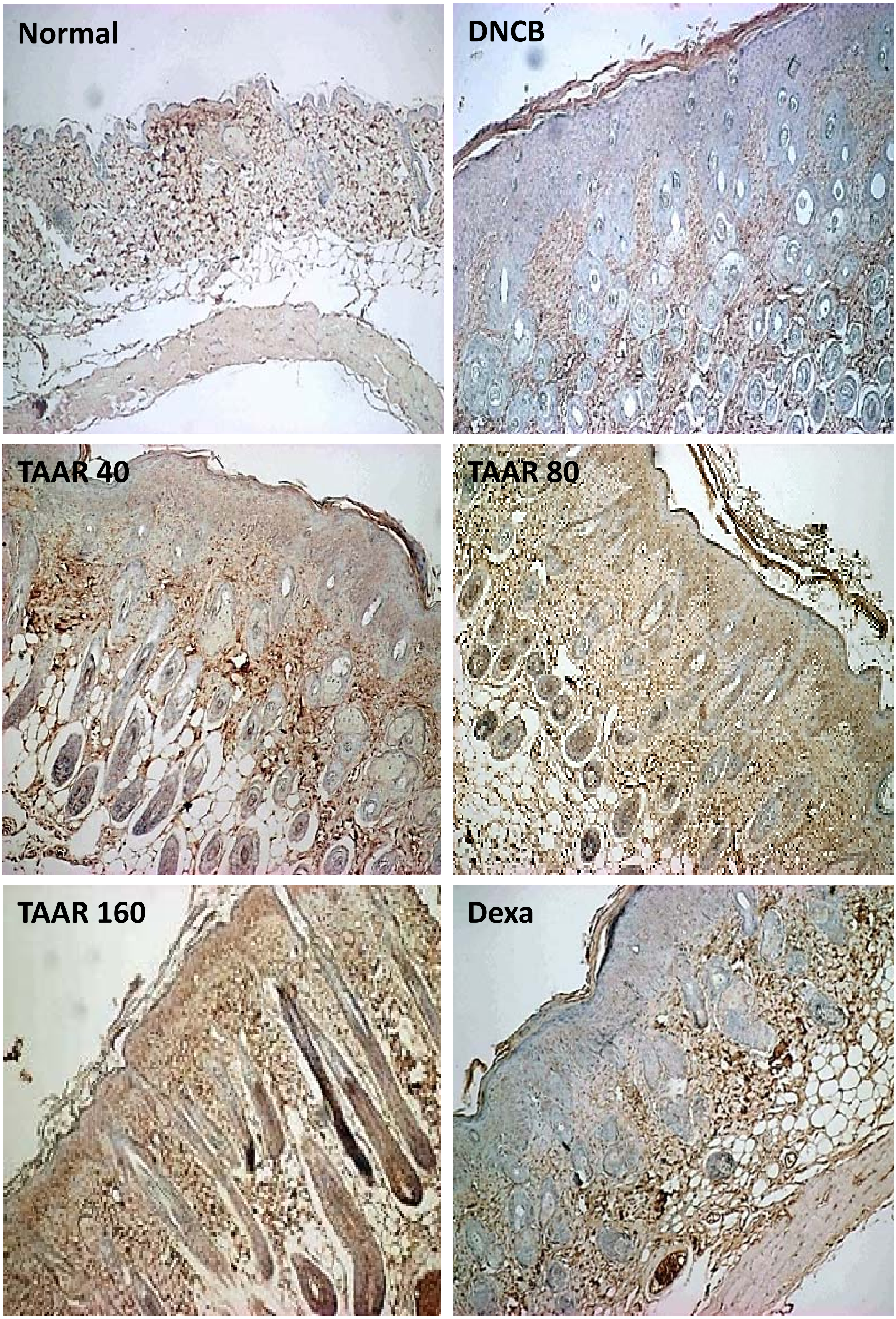

Supplement: Supplementary file 1 [file antioxidants-12-00027-s001.zip › antioxidants-2008242-supplementary.pdf]
